# Educator‐Led Screening to Identify 4‐Year‐Old Children At‐Risk of Motor Delay: A Mixed Methods Convergent Study

**DOI:** 10.1111/cch.70314

**Published:** 2026-07-20

**Authors:** Laura Brown, Amanda Bacon, Annie Chappell, Verity Pacey, Nicole Pates, Emre Ilhan

**Affiliations:** ^1^ School of Health Sciences and Nursing, Faculty of Medicine, Health and Human Sciences Macquarie University Sydney Australia; ^2^ Movement Mechanics Physiotherapy Brisbane Australia; ^3^ Western Kids Health Perth Australia; ^4^ School of Human Movement and Nutrition Sciences University of Queensland Brisbane Australia

**Keywords:** developmental outcomes, education, educator, motor delay, screening

## Abstract

**Background:**

Developmental screening can assist in identifying children ‘at‐risk’ of developmental delay. The childcare setting may be the ideal location for screening most children for developmental delay. Therefore, our aim was to determine the feasibility of early childhood educator‐led Targeted Motor Control (TMC) screening to identify 4‐year‐old children ‘at‐risk’ of motor delay.

**Methods:**

In this study, conducted in Australia, we used a mixed methods convergent design. Educators attended a 2‐h in‐person TMC training, which included education, and a demonstration and scoring video that educators watched and scored, respectively. Educators then screened a minimum of four children each aged between 3 years 9 months to 4 years and 5 months, using the TMC. The final TMC that each educator performed was coscored by an experienced TMC‐trained physiotherapist. Feasibility of the training and use of the TMC was evaluated using an online survey completed by all educators that covered five feasibility themes: acceptability, demand, practicality, implementation and integration. The survey, comprising closed and open‐ended questions, was completed by educators after performing all TMC screening.

**Results:**

Thirteen educators screened four children each and completed the survey. In terms of major findings, merging of quantitative and qualitative data demonstrated convergence for the themes of acceptability, demand and implementation with 100% agreement between educator and physiotherapist scoring on the TMC. However, there was divergence of findings for the feasibility themes of practicality and integration.

**Conclusion:**

It can be concluded that it may be feasible for early childhood educators to perform TMC screening, but we recommend modifications to the training and resources to enable educators to perform screening with greater success.

## Summary

- Early identification of developmental delay is recommended.
- Developmental screening can assist in detecting mild developmental delay where the delay is subtle and may be difficult to identify.
- Early childhood educators can perform Targeted Motor Control (TMC) screening to identify 4‐year‐old children ‘at‐risk’ of motor delay if equipped with the appropriate training, resources and support from physiotherapists.
- Further modifications of TMC training, resources and support have the potential to enhance and expand the use of TMC by nonhealth professionals.

## 1 Introduction

Developmental delay is a term that describes development that is slower to progress than what is typically expected (Council on Children With Disabilities et al. 2006; Whitehouse 2023). Developmental delay is often used for children under 5 years of age as it is considered a temporary state in child development (Whitehouse 2023). Children may experience developmental delay in a single domain, such as communication, motor, social, emotional and/or cognitive skills, or in multiple domains (known as global developmental delay), and this delay may have associated activity limitations and participation restrictions for affected children (American Psychiatric Association 2022). In Australia in 2024, 23.5% of children experienced a number of challenges in developmental domains and were ‘developmentally vulnerable’, and only 52.9% of children were ‘developmentally on track’ (Australian Government Department of Education 2024). If a developmental delay persists, a developmental disability may result. Persons with disabilities include those who have ongoing physical, mental, intellectual or sensory impairments, which may limit their ability to participate fully and effectively in everyday life on an equal level with others (United Nations 2006). Consequently, early identification of developmental delay is recommended (Lipkin et al. 2020). Early identification of delay allows children to be referred to early intervention, if indicated, so that a child’s long‐term outcomes can be optimised (Lee and Zwicker 2021; Blank et al. 2019; Williams and Holmes 2004).

When a child has a delay in the attainment of motor skills, this is known as motor delay. This means that the development of age‐appropriate gross motor and fine motor skills is behind that expected for age (Van der Walt et al. 2020). In Australia in 2024, 10.0% of children were ‘developmentally vulnerable’ on the physical health and well‐being domain, which includes gross and fine motor skills (Australian Government Department of Education 2024). The development of motor skills provides foundations for early learning and progression, which is critical to future success both in an academic and sporting context (Veldman et al. 2020). Motor development is also important for overall health outcomes, such as maintaining a healthy weight (Lubans et al. 2010; Veldman et al. 2016). The consequences of motor delay include difficulties performing activities of daily living, poor academic performance, difficulty meeting physical activity guidelines and struggles with peers (Engel et al. 2018; Lubans et al. 2010; Robinson et al. 2015).

A critical timepoint for identifying a delay, such as a motor delay, is prior to children commencing full‐time school. One reason for this is so that school readiness can be determined to promote a smooth transition into formal education (Lipkin et al. 2020). Motor skills are particularly relevant prior to school entry, as they have been shown to predict school readiness (Jones et al. 2021). In Australia, formal childcare provides early childhood education and care for children up to the age of 5 years (Australian Government Department of Education 2023). Therefore, 4 years of age, when many children are attending some form of childcare, may be the optimal time to identify developmental delay using a standardised developmental screening test (Lipkin et al. 2020). Additionally, the brain is still considered very plastic or responsive to change at 4 years of age, so if a delay is identified, it is an optimal timepoint to intervene (Blauw‐Hospers and Hadders‐Algra 2005; Sices 2007).

Screening, involving the use of a simple and quick tool, can assist educators and teachers in identifying children with developmental delay, including motor delay (Lipkin et al. 2020). According to the World Health Organisation (WHO) (Wilson et al. 1968), screening is important for children who do not show obvious signs of impairments but have underlying mild developmental delay where the delay or developmental deviation is subtle and may be difficult to identify (Williams and Holmes 2004). Early screening is particularly important given that up to one‐half of children with developmental delay will remain undetected before 6 years of age (Mackrides and Ryherd 2011). One factor contributing to this lack of identification of developmental delay may include reliance exclusively on surveillance methods (checklists and clinical observation) (Mackrides and Ryherd 2011). Additionally, relying on health professionals in healthcare settings to screen is not practical due to barriers such as cost, time pressures, staffing needs, poor agreement on the most suitable tool to use and competing clinical demands (Mackrides and Ryherd 2011). A pragmatic way to establish routine screening for 4‐year‐old children is in the childcare setting.

In 2017, 58% and 45% of all 3‐ and 4‐year‐old children, respectively, attended formal childcare or a combination of formal and informal care in Australia (Australian Bureau of Statistics 2018). Early childhood education and care is delivered by qualified educators through a formalised learning framework (Australian Government Department of Education 2023). Therefore, early childhood education and care centres may be the ideal location for screening most children for developmental delay, such as motor delay. Additionally, early childhood educators and teachers, who will collectively be referred to as educators throughout this study, spend large amounts of time with 4‐year‐old children attending their centres, so they may be able to facilitate the early identification of delay through the use of a screening tool. This would mean educators could screen a large number of children in a potentially more cost‐effective and efficient manner than health professionals performing screening. There are acceptable (in terms of specificity and sensitivity) questionnaire‐based screening tools that can identify developmental delay (Sheldrick et al. 2020; Van Dokkum et al. 2022), such as Ages and Stages Questionnaire, Third Edition and Parent’s Evaluation of Developmental Status. However, performance‐based screening tools have been shown to be superior to questionnaires for detecting motor problems as they may provide a more objective view of the child’s performance (Van Dokkum et al. 2022). There is a lack of performance‐based screening tools that screen for both motor and sensory skills and that can be used in the community setting (Van Dokkum et al. 2022).

The Targeted Motor Control (TMC) screening tool (developed by authors of this study), which is a performance‐based test, may allow educators to identify children at‐risk of developmental delay, in particular, motor delay. The TMC has been validated (Brown et al. 2024) against the Neurosensory Motor Developmental Assessment (NSMDA) (Burns 2014). In the TMC validation study, involving 76 children, the authors found that there were significant and positive moderate correlations between the item totals overall and for each area on the NSMDA and the TMC and between NSMDA functional grade for each area and the corresponding TMC areas (Brown et al. 2024). TMC is an 11‐item screening tool that takes approximately 15–20 min for physiotherapists to administer. Although the TMC was developed to identify children at‐risk of motor delay (gross motor, fine motor and sensorimotor such as visual or tactile concerns), it may indirectly detect children at‐risk of delay in other developmental domains. The sum of the 11 items gives a total TMC score, ranging from −2 to 11. Children who score less than 9/11 are ‘at‐risk’ of atypical motor development and further assessment should be considered. Children who score 9/11 or higher are considered to be within the normal range for age. Details on how the TMC score was validated to determine children at‐risk of motor delay when performed by physiotherapists can be found in Brown et al. (2024).

Resources have been developed to guide TMC users. An instructional manual was created to guide physiotherapists. Additionally, training and resources have been developed to improve the feasibility of using the TMC by nonhealth professionals (Hami et al. 2025).

Therefore, in this study, our aim was to answer the following question: Is it feasible for early childhood educators to perform TMC screening to identify 4‐year‐old children at‐risk of motor delay?

## 2 Methods

### 2.1 Study Design

A convergent mixed methods design was used in this study. Quantitative data and qualitative data were collected in a single‐phase approach, analysed separately and then compared during the interpretation phase (Creswell and Creswell 2018). A mixed methods approach allowed for multiple perspectives to be developed and a complete understanding of the research question to be reached.

### 2.2 Ethical Considerations

This study was approved by the Macquarie University Medicine and Health Sciences Subcommittee (reference number: 520231317647110).

### 2.3 Recruitment Strategy

Recruitment occurred during July and August 2023 in two major cities in Australia, Brisbane and Perth. A convenience sampling method was used. Information flyers about the study, which included eligibility criteria and contact details, were distributed to early childhood education and care centres (collectively referred to as childcare centres in this study) via email and social media platforms (such as Facebook and Instagram). Childcare centres facilitated sharing the information flyers with parents/caregivers of eligible children at their centres. Eligible educators and parents/caregivers of eligible children were provided with participant information and consent forms. There were separate written consent forms for educators and parents/caregivers of children. Information in the forms included details about the study, steps involved in participating, potential risks, data management and how to seek further information. It was also clear in these forms that the study was voluntary with an option to withdraw at any time. It was anticipated that 5–10 educators and approximately 20–30 children would be recruited to the study. This sample size was based on logistics and what the research team believed was realistic in terms of recruitment of educators and children within the designated timeframe. It was also based on how much practice the research team anticipated that the educators would need before the physiotherapist coscored with them to determine how accurate they were with their scoring.

### 2.4 Participants

Educators were eligible to participate in the study if they were an early childhood educator or teacher as recognised by the Australian Children's Education and Care Quality (ACECQA) standards. Additionally, educators had to work in a childcare facility within a 1‐h drive from Brisbane or Perth Central Business District and had to have access to between four and five eligible children at their facility. Furthermore, educators had to have internet access and needed to be able to speak and understand English. Educators were excluded if they did not hold an approved diploma level qualification (or similar, if from outside Australia). Also, educators were excluded if they were unable to perform any TMC items, despite the addition of manual handling modifications. For example, one item, horizontal head righting, involved a manual handling component where educators were required to lift the child. However, this item could be modified by educators putting their foot on a chair and tipping the child over their knee to perform the item.

Children were eligible to participate in the study if they were aged between 3 years 9 months and 4 years and 5 months, they attended formal centre‐based childcare, they were willing to undergo testing of the TMC by an educator, their parent provided informed consent to be a part of the study, and they had access to an eligible educator at their facility. Children also needed to be able to speak and understand English. Children were excluded if they had already been diagnosed with a moderate to severe developmental delay or intellectual or behavioural disability that meant they could not complete any item on the TMC. For example, children were excluded if they had confirmed neurological impairments, a visual impairment not corrected by wearing corrective lenses or a hearing impairment not corrected by aids.

### 2.5 Procedures

Once consent was obtained, educators underwent group‐based TMC training delivered face‐to‐face by two physiotherapists at each site who were involved in developing the TMC (these two physiotherapists were also authors of this study). TMC training occurred in Brisbane at an early learning centre (pre‐prep) and in Perth at a centre that provides paediatric allied health services in the community. The training was approximately 2 h and included an initial presentation about the TMC, a TMC demonstration video, a TMC scoring video that educators used to check their competency in using the screening tool, then an opportunity for discussion and questions. Educators were advised that they could use verbal cueing or verbal instructions as required when performing TMC screening to ensure that the child undergoing testing understood what they were required to do for a particular item. For example, for horizontal head righting (eyes open), educators could ask the child to focus on something and to stand tall. For the scoring video, if there was a lack of congruence in the educators’ interpretation of the test with the correct interpretation (according to the team that developed the TMC), the plan was for these educators to rewatch the demonstration and scoring video and then score and interpret this latter video again. After completing the training and correctly interpreting the TMC scoring video, educators had ongoing access to the training resources, as well as a website that included the TMC manual and score sheet. They also had access to an online forum where they could post questions that the research team would answer.

Educators then assessed children who met the eligibility criteria and whose parents/caregivers consented to their child being involved in the study. Each educator assessed four children. We anticipated that practice with using the TMC would have a positive impact on educators’ perspectives on feasibility. One of the physiotherapists who delivered the TMC training coscored one TMC assessment per educator. Educators assessed three children before this coscoring occurred. The coscoring ensured that all aspects of feasibility were addressed.

Following completion of TMC assessments, feedback was collected from educators via an online survey through Qualtrics (see Appendix 1 for full version of survey). The survey asked about demographic information and feasibility with using the TMC following the training.

General feedback was collected from the two physiotherapists who led the TMC training and who coscored the TMC educators via a one‐page reflection (Appendix 2). This reflection included details on what the physiotherapists felt worked well and what required modifications following the training and observation of the educators performing the TMC assessments.

In cases where a child had an ‘at‐risk’ result on the TMC according to the educator and, following discussion with the physiotherapist, it was decided that the child’s performance was of concern, a report was written by the physiotherapist. This report was provided to the child’s parents/caregivers notifying them of the concern and offering them an opportunity to talk to a physiotherapist involved in the study. The purpose of this discussion was for the physiotherapist to discuss the child’s results with the parents/caregivers and to guide them regarding appropriate follow‐up services for their child. Educators were given guidelines to help with determining whether a child’s result was concerning, including: a score of less than 9 = consider further assessment, score equal to or greater than 9 = no further assessment needed.

If any parent/caregiver requested information about their child’s motor performance, the research team provided a brief summary.

### 2.6 Measurements

#### 2.6.1 Primary Outcome Measure

##### 2.6.1.1 Feasibility Measures

The concepts of feasibility were measured via an online survey, quantitatively using a 5‐point Likert scale, from strongly disagree to strongly agree with a neutral option, and qualitatively using open‐text questions.

Feasibility was operationalised using Bowen et al.'s framework (with questions framed accordingly) to determine how feasible the TMC was following the training session, ongoing support by the physiotherapist and the availability of the online resources (Bowen et al. 2009). Members of the research team reviewed the survey and minor changes were made prior to its distribution. Feasibility domains included acceptability, demand, implementation, practicality and integration. Appendix 3 provides further detail about the operationalization of the domains including mapping of Bowen et al.'s feasibility framework (Bowen et al. 2009) with quantitative and qualitative questions.

#### 2.6.2 Other Outcome Measures

Demographic information about educators was collected via the survey. As part of this, educators were asked to rate their confidence in identifying 3–4‐year‐old children with mild developmental delay on a scale from 0 to 10 (0 = no confidence, 10 = full confidence). Given the inclusion criteria for children in the study was 3 years 9 months to 4 years 5 months, the question asked about 3–4‐year‐old children.

### 2.7 Data Analysis

All quantitative data was analysed descriptively using median (range), frequencies and percentages. For example, when analysing educators’ confidence in identifying 3–4‐year‐old children with mild developmental delay, median (range) was provided. Level of agreement in total TMC scores between educator and physiotherapist was determined using a Bland–Altman plot. Qualitative data from the open‐text responses in the survey was summarised by the Chief Investigator (CI) who then used a deductive thematic approach to analyse responses according to Bowen et al.'s feasibility framework (Bowen et al. 2009). The summaries and analysis performed by CI were cross‐checked for accuracy by a second member of the research team to ensure that they captured the key points of the feedback provided by educators according to the framework.

The merging of quantitative and qualitative data was done via the joint display of data (Creswell and Creswell 2018; Aschbrenner et al. 2022). This allowed for an integrative data analysis, where comparisons were performed between the quantitative and qualitative data for each domain (e.g., acceptability) so that conclusions could be drawn. Meta‐inferences were made to determine alignment of quantitative and qualitative data.

Statistical analyses were conducted using Microsoft Excel for Mac (Microsoft Corporation, version 16.79.2) and GraphPad Prism version 10.1.1 for Mac (GraphPad Software, Boston, Massachusetts USA).

## 3 Results

### 3.1 Demographic Findings

Fourteen educators from four different centres were recruited and underwent TMC training. No educators were excluded due to an inability to perform any TMC items. One educator withdrew from the study after screening two children due to personal commitments unrelated to the study. Thirteen educators screened four children each. All 13 educators completed the feedback survey. One child withdrew from the study after the screening had occurred (parent/caregiver request). Therefore, 13 educators and 51 children were included in the study (Appendix 4).

No questions were posted on the TMC Q and A forum during the study. Reports, including recommendations, were written for 16% children (*n* = 8) due to concerning TMC results and following discussion about the child’s result with the attending physiotherapist. These reports were passed on to parents/caregivers.

All educators described their gender as woman or female and 77% (*n* = 10) had more than 10 years' experience working in the childhood setting (Table 1).

**TABLE 1:** Demographic details of educators (*n* = 13).

**Age, *n* (%)**	
20–30 years	2 (15.4)
31–40 years	3 (23.1)
41–50 years	4 (30.1)
51–60 years	1 (7.7)
>60 years	3 (23.1)
**Gender, *n* (%)**	
Female	13 (100.0)
**Place of work, *n* (%)**	
Queensland	6 (46.2)
Western Australia	7 (53.8)
**Primary role at workplace, *n* (%)**	
Childhood educator/teacher/leader	11 (85.0)
Administrator	1 (7.7)
Other—educational leader	1 (7.7)
**Employment status, *n* (%)**	
Full‐time	11 (85.0)
Part‐time	2 (15.4)
**Highest academic qualification, *n* (%)**	
Bachelor of education (early childhood)	9 (69.2)
Diploma of early childhood education and care	1 (7.7)
Master of education	1 (7.7)
Master of teaching (early childhood)	1 (7.7)
Bachelor of science speech and hearing	1 (7.7)
**Length of time working in early childhood, *n* (%)**	
Between 3 and 5 years	3 (23.1)
>10 years	10 (77.0)

When educators were asked to rate their confidence in identifying 3–4‐year‐old children with mild developmental delay on a scale from 0 to 10 (0 = no confidence, 10 = full confidence), the median was 7 (range = 6–9). All educators had experience working with children with developmental delay and all educators, except one, reported they had previously identified children with developmental delay. Strategies used to identify children with developmental delay included: observations (77.0%), knowledge of age‐appropriate development (30.8%), collaboration with colleagues and health professionals (53.8%), developmental checklists/screening tools (53.8%) and Fundamental Movement Skills programme (15.4%). Fundamental Movement Skills are considered the foundation movements for higher level movement sequences needed for a range of sport and recreational activities and include locomotor and object control skills (Lubans et al. 2010).

Of the 51 children screened, there were 18 males and 30 females. The gender of three children was not recorded. When the TMC was performed by the educators, 34 children scored less than 9/11 on the TMC and the mean score for all children screened using the TMC was 7 (SD = 2.4). Eleven out of the 13 educators had a mix of children who, based on the TMC, were classified as ‘at‐risk’ or within typical range for age. Two educators only screened children who ended up with an ‘at‐risk’ outcome on the TMC.

### 3.2 Feasibility Data

Quantitative and qualitative findings from the survey have been presented via joint display of data for each domain: acceptability (Table 2), demand (Table 3), implementation (Table 4), practicality (Table 5) and integration (Table 6). An integrative analysis based on comparisons between the quantitative and qualitative data for each domain is presented in the next section. Where appropriate, any divergence in findings between the quantitative and qualitative data has been highlighted.

**TABLE 2:** Joint data display of quantitative and qualitative data for the domain of acceptability.

Operationalization of acceptability	Quantitative data summary	Qualitative data summary
The fit of the TMC tool within the organisational culture of the early childhood care centre. The perceived appropriateness of the TMC tool. The intention for ongoing use of the TMC tool. Satisfaction with the TMC training and use of the TMC tool	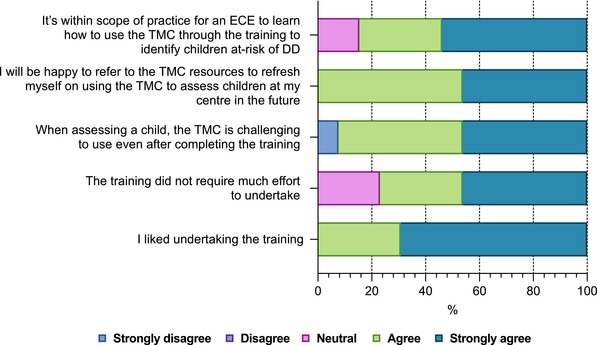	Educator 1 (> 10 years' experience, Brisbane) ‘ … it was good to have the manual to refer to …’. Educator 13 (> 10 years' experience, Perth) ‘I would have preferred to watch a few more assessments before doing them’. Educator 7 (3–5 years' experience, Perth) ‘[training should] allow for more practices of implementing and sorting’.

Abbreviations: DD = developmental delay; ECE = early childhood educator; TMC = targeted motor control.

**TABLE 3:** Joint data display of quantitative and qualitative data for the domain of demand.

Operationalization of demand	Quantitative data summary	Qualitative data summary
Perceived demand for the TMC training and TMC tool for self and others. Perceived demand for further training	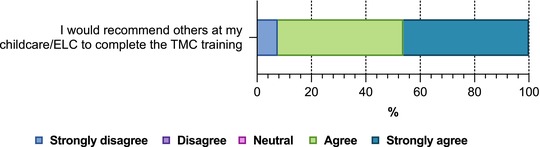	Educator 6 (> 10 years' experience, Brisbane) ‘We would love to learn more on how to support children in the classroom environments after the assessment’. Physiotherapist 1 (Brisbane) ‘Educators are keen for more knowledge about the interpretation of results’. Educator 2 (3–5 years' experience, Brisbane) ‘More info about how to talk with parents/caregivers about the screening’.

Abbreviations: DD = developmental delay; ECE = early childhood educator; TMC = targeted motor control.

**TABLE 4:** Joint data display of quantitative and qualitative data for the domain of implementation.

Operationalization of implementation	Quantitative data summary	Qualitative data summary
Degree of success of the TMC training to enable use of the TMC tool by educators. Efficiency, speed or quality of using the TMC tool	Nonsurvey data 100% of educators provided the correct recommendation (i.e., at‐risk of atypical development and further assessment should be considered or within the normal range for age) for the TMC scoring video. 100% agreement between educator and physiotherapist scoring on the TMC, across all subarea scores and total score. 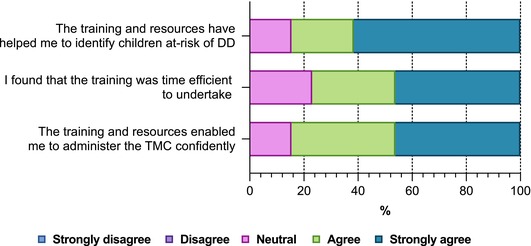	Educator 13 (> 10 years' experience, Perth) ‘[I] found it difficult to score the negative questions. I should have watched the training video a few more times. I will make sure I watch it before I do any more’. Educator 2 (3–5 years' experience, Brisbane), Educator 3 and Educator 5 (both > 10 years' experience, Brisbane) all reported they encountered ‘nil’ problems when using the TMC to assess children after completing the training programme. Educator 11 (> 10 years' experience, Perth) ‘… having a negative score for 2 [items] could be confusing’. Educator 1 (> 10 years' experience, Brisbane) ‘We like that the scores are 0, 1 or −1. Any more than this would be confusing’. Physiotherapist 2 (Sydney) ‘Staff needed the physio support for at least the first couple of assessments … lots of feedback was needed for staff for ocular motor and tactile [items]’.

Abbreviations: DD = developmental delay; ECE = early childhood educator; TMC = targeted motor control.

**TABLE 5:** Joint data display of quantitative and qualitative data for the domain of practicality.

Operationalization of practicality	Quantitative data summary	Qualitative data summary
Ability of educators to use the TMC tool after undertaking the training with the existing resources, time and/or commitments.	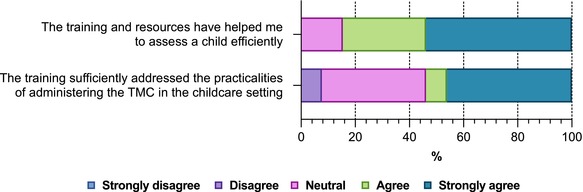	Educator 7 (3–5 years' experience, Perth) ‘It was difficult to score in between tasks as 3‐4 year‐olds lose patience and energy very quickly’. Educator 11 (> 10 years' experience, Perth) ‘Lifting and tilting students could be tricky if students are heavy, tall or teachers have back/knee health issues’. Physiotherapist 1 (Brisbane) ‘Verbal cues for Head Righting task would be helpful’. Educator 1 (> 10 years' experience, Brisbane) ‘We were worried about lifting and tipping—this was much easier than I thought’.

Abbreviations: DD = developmental delay; ECE = early childhood educator; TMC = targeted motor control.

**TABLE 6:** Joint data display of quantitative and qualitative data for the domain of integration.

Operationalization of integration	Quantitative data summary	Qualitative data summary
The perceived fit of the TMC training and TMC tool within the existing infrastructure of the centre. Perceived sustainability of the TMC training and TMC tool	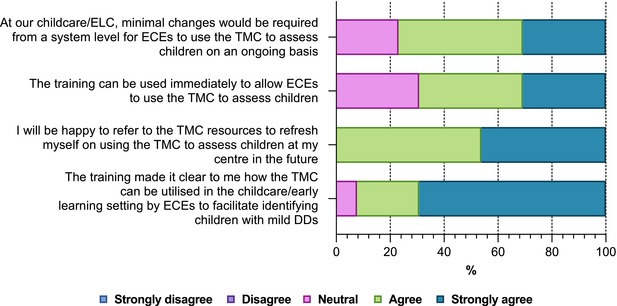	Educator 7 (3‐5 years' experience, Perth) ‘Allow for more time (schools need to value how long it takes when first learning it). Consider teachers who are neurodiverse and may take a little longer to understand the scoring’. Educator 10 (3‐5 years' experience, Perth) ‘This programme would be beneficial for schools that do not have access to onsite [Occupational Therapists] who already screen the children’.

Abbreviations: DD = developmental delay; ECE = early childhood educator; TMC = targeted motor control.

### 3.3 Integrative Analysis

#### 3.3.1 Acceptability

The findings from the joint display highlight that although the training and support was enough to begin using the TMC, that in order for the training and the use of the TMC by educators to be more acceptable, ongoing support may be ideal to build confidence and independence. Additionally, although the quantitative data indicates that it is suitable for educators to perform the TMC, qualitative data did not touch on this part of acceptability.

#### 3.3.2 Demand

Although TMC training was considered to be a worthwhile undertaking, there was an additional demand by educators to learn (1) how to communicate TMC findings to families and (2) what to do in terms of supporting children identified as ‘at‐risk’ on the TMC following the screening. Information on how to discuss findings with families and how to support children is not available in the current training package. The physiotherapists also identified that educators were interested in learning more about how to interpret the results. In the qualitative data, educators did not provide any comments about recommending the TMC training to others or actually using the TMC in practice.

#### 3.3.3 Implementation

Educators were capable of performing TMC screening successfully following completion of the training and with the support from the physiotherapist with their initial two to three assessments. Most educators believed that the in‐person training, resources and support enabled them to administer the TMC. However, educators reported ongoing difficulties with recalling the scoring and interpretation of the items in the TMC. The physiotherapists recognised that educators required the support of the physiotherapists when initially performing the TMC as well. This reinforces the potential of enabling practice during training and/or providing more support following training.

#### 3.3.4 Practicality

Educators felt that the training and resources enabled them to perform TMC screening efficiently, but it was difficult for them to do the screening due to the age of the children and other demands of educators’ daily schedule. This suggests the need for staff support for sustainability of TMC screening by educators. Although concerns were raised by some educators about performing one item (horizontal head righting), these concerns were not shared by others when strategies to reduce manual handling burden were provided, as well as verbal cues to be used with the child on this item. The physiotherapists also noted that verbal cues for this item would be useful.

#### 3.3.5 Integration

Following the training and with ongoing access to the TMC resources, educators felt that they could use the TMC to identify children ‘at‐risk’ of developmental delay. Consideration of the needs of different educators was emphasised, such as for neurodiverse educators who may require extra time for training and using the TMC. From an organisational level, the TMC requires minimal changes for educators to use it on an ongoing basis, and it was felt that this form of screening is beneficial in centres that do not already screen children.

### 3.4 Areas for Improvement in Future Iterations of the TMC

There were four areas suggested for improvement of the training: (1) making it clear during the training that more than one demonstration can be provided to children; (2) further simplifying the language in the manual for educators; (3) pausing videos during training to highlight items and techniques for administering the item, discuss the child's performance and how the child scored, especially for the latter, more difficult items; and (4) creating videos just for scoring, labelling the items presented in the videos with the item numbers on the scoring sheet. See Appendix 5 for further details on areas for improvement.

There were two areas suggested for improvement of the TMC tool: (1) a numbered scale on the scoring sheet to identify which items are scored from 0 to 1 and −1 to 1 and (2) state the required numbers (e.g., number of hops) needed for each skill to be considered met/age‐appropriate.

## 4 Discussion

This convergent mixed methods study demonstrated that following TMC training and with initial support from physiotherapists, it is feasible for early childhood educators to perform TMC screening to identify 4‐year‐old children ‘at‐risk’ of motor delay. However, we recommend modifications to the training and resources to enhance the use of the TMC by educators, particularly in relation to practicality and integration.

The results of the joint display demonstrate convergence of quantitative and qualitative findings for acceptability, demand and implementation. Acceptability was affirmed as educators liked the TMC training, it took minimal effort to undertake, and they believed and demonstrated that screening was within their scope of practice. Educators indicated that the training and support, in its current form, should be the minimum amount. This is interesting given most (10/13) educators in the study had more than 10 years' experience working in the childcare setting and all educators in the study had worked with children with developmental delay. The suitability of educators performing the screening is not surprising given, for some time now, educators have been utilising surveillance questionnaires in the childcare setting (Filgueiras et al. 2013), and it has been shown that educators are able to identify children ‘at‐risk’ of developmental delay (Branson 2009) or concern (Kiing et al. 2019). More recently, it has been reported that childcare providers believe that screening is part of their role and approximately one‐fifth already perform screening on a routine basis (Boh and Johnson 2018). Demand was demonstrated in the quantitative data by almost all educators recommending the TMC training to their colleagues, although this was not mentioned in the qualitative data. Furthermore, there was an additional demand for further education about what the TMC results mean and this was identified by the physiotherapists as well. Additionally, educators wanted to know how they can help ‘at‐risk’ children. This finding in relation to demand and how educators want to play an active role in identifying motor developmental delay is consistent with literature indicating that most teachers believe they should be involved in identifying early warning signs of disorders such as developmental co‐ordination disorder (Hunt et al. 2021). The joint display findings regarding implementation showed that the TMC training and resources enabled educators to perform TMC screening accurately and effectively even though some educators found recalling items difficult and they required the support of the physiotherapist initially.

There was some divergence between quantitative and qualitative findings for the feasibility themes of practicality and integration. In terms of practicality, although the educators found that the TMC training and resources allowed them to perform the screening efficiently, the challenging age of the children and the busyness of the daily schedule impacted on feasibility in this area. A solution to this practical concern is to provide educators with teaching support so that it is more manageable for them to perform the screening because it would add another responsibility to their workload. Ensuring that there is ‘enough relief staff to cover’ so that staff can take time to be trained in the use of screening tools and then perform screening has been promoted in a study exploring barriers and facilitators to implementation of developmental screening (Peterson‐Katz et al. 2023). This study included early learning and care and education staff. However, additional staff may not be financially viable within a stand‐alone centre. Therefore, in the long term, supporting educators to perform screening may require a unified national government‐funded approach. There was agreement that following the TMC training and with ongoing access to the TMC resources, screening by educators could be integrated into the childcare setting, but, in doing so, there needs to be consideration of the individual needs of each educator. Additionally, if the suggestions for improvement to the TMC training and the TMC tool are actioned, this would enhance the usability of the TMC.

The consistencies and inconsistencies that we have found between the quantitative and qualitative data in our study have enabled us to gain insight about feasibility and have also helped us to identify implications for future work. If the recommendations are implemented to address the challenges associated with practicality and integration, then another study could be conducted to examine the feasibility of educators performing TMC screening on a much wider scale, such as across multiple centres to determine generalisability. This could lead to deeper insights and stronger conclusions regarding the feasibility of the training, the TMC tool and an educator‐led screening programme for mild motor delay. Additionally, our study has shown that it is feasible for educators to perform screening, which provides a conduit to routine screening becoming a reality prior to children commencing formal education. Indeed, such an initiative has wider public health implications – a vision for routine universal screening would align with current recommendations based on guidelines from the American Academy of Pediatrics (Lipkin et al. 2020). The next consideration then is how to manage children identified as ‘at‐risk’ of developmental delay on screening, as we know that early referral to appropriate services may optimise outcomes for these children (Blank et al. 2019). This process could involve training educators on how to communicate with parents/caregivers about screening results and referral pathways, as well as developing and trialling an intervention model that includes input from stakeholders (including educators, health professionals and parents). In this way, educator‐led developmental screening combined with evidence‐based and meaningful intervention for children with developmental delay could improve outcomes and later school participation for these children.

There are several strengths and limitations of our study. The finding that it is feasible for educators to perform screening has clinical significance as it provides an efficient approach to routine screening of children. However, we do acknowledge that our sample size of 13 is small so caution needs to be taken with generalisability of our findings, especially as most of the educators in our study had greater than 10 years' experience working in the childcare setting. The TMC training and use of the TMC may be different for less experienced users. Another limitation relates to an element of bias in our sample population. Educators in our study were from privately funded centres and were highly motivated and keen learners, which may not be representative of educators at large. Additionally, there may have been potential bias in our study in terms of including children in which there were developmental concerns. Involvement in the study provided a free opportunity to objectively confirm motor concerns, and it assisted in next steps to assist children identified as at‐risk. This likely explains the high number of children identified as at‐risk on the TMC in this study (34 out of 51). Finally, there were only two open‐ended questions to derive qualitative data. Although this meant that responses could fall under any of the feasibility domains, educators were not prompted to provide specific feedback on each domain. However, educators included a lot of detail in their responses to these questions which provided depth and allowed coding of their responses according to feasibility themes.

### 4.1 Conclusions

Our study aimed to investigate whether it is feasible for early childhood educators to perform TMC screening to identify 4‐year‐old children at‐risk of motor delay. Using a mixed methods approach, we have demonstrated that it is feasible, but we recommend modifications to the TMC training and resources to improve the application and integration of the screening into the childcare setting. The results from our study are promising and future work should build on our findings to ensure early identification of children with developmental delay, especially motor delay, by educators.

## Author Contributions

There are six authors on this manuscript and each author meets authorship criteria established by the International Committee of Medical Journal Editors (ICMJE). Authors on this manuscript meet all three of the following criteria: substantial contributions to the conceptions and design, or acquisition, analysis or interpretation of data; drafting the article or revising it critically for important intellectual content; final approval of the version to be published.

**Laura Brown:** conceptualisation, data curation, formal analysis, funding acquisition (OSP), investigation, methodology, project administration, software, supervision, validation, visualisation writing – original draft preparation, writing – review and editing. **Amanda Bacon:** conceptualisation, investigation, resources, writing – review and editing. **Annie Chappell:** investigation, resources, writing – review and editing. **Nicole Pates:** investigation, resources, writing – review and editing. **Verity Pacey:** conceptualisation, formal analysis, methodology, visualisation, writing – review and editing. **Emre Ilhan:** conceptualisation, data curation, formal analysis, methodology, software, validation, visualisation, writing – original draft preparation, writing – review and editing.

## Funding

This study received no external funding but was supported by an Outside Studies Program (OSP) Research Fellowship. L.B. was awarded this Fellowship and this provided a grant of salary for a 3‐month period for L.B. to undertake research and scholarship activity relevant to this study.

## Ethics Statement

This study was approved by the Macquarie University Medicine and Health Sciences Subcommittee (reference number: 520231317647110).

## Consent

Participants provided informed written consent to participate in this study. Informed consent for publication was provided by all participants.

## Conflicts of Interest

Amanda Bacon developed the TMC in 2011 and has been using the TMC screening tool within her private business since 2011. Laura Brown and Amanda Bacon are in the process of increasing the reach of the TMC screening tool and accrediting health and nonhealth professionals for a fee. A.C., E.I., N.P. and V.P. have no conflicts of interest to declare.

## Data Availability

Data for this study are available upon request due to privacy/ethical restrictions.

## Appendix 1 Survey

Qualtrics survey: post‐training feasibility and feedback questionnaire.

### Demographic questions

1 What is your name?
2 What is your age?

○ 20–30 years
○ 31–40 years
○ 41–50 years
○ 51–60 years
○ > 60 years

3 How do you describe your gender?

○ Woman or female
○ Man or male
○ Nonbinary
○ I use a different term (please specify)
○ Prefer not to answer

4 What is your primary role at the Childcare/Early Learning Centre?

○ Childhood educator/teacher/carer
○ Administrator
○ Health professional

5 Which of the following categories best describes your employment status?

○ Full‐time employee
○ Part‐time employee
○ Casual employee

6 How long have you worked in early childhood?

○ < 3 months
○ < 1 year
○ Between 1 and 2 years
○ Between 3 and5 years
○ Between 6 and 10 years
○ > 10 years

7 What is the highest academic qualification that you hold:

○ Certificate III in Early Childhood Education and Care
○ Diploma of Early Childhood education and Care
○ Bachelor of Education (Early Childhood and Primary)
○ Other ___________________________

8 How would you rate your confidence in detecting mild developmental delay in children 3–4 years old (0 [no confidence at all] to 10 [full confidence])?
9 Have you ever worked with or had experience looking after children with developmental delay?

○ Yes
○ No
○ I don’t know what developmental delay is

10 Have you previously identified children with developmental delay?

○ Yes
○ No
○ I don’t know what developmental delay is

11 If you answered ‘Yes’ to question 10, what strategies did you use to identify children with developmental delay?

**Questions related to the feasibility of the training programme**

12 To what extent do you disagree or agree with each of the following statements:

Strongly disagree
Disagree
Neutral
Agree
Strongly agree

Note: ‘resources’ include TMC website with manual, score sheet and question/answer forum, as well as ongoing access to the TMC information and scoring videos.

**Table:**

Component	Statement	1	2	3	4	5
Acceptability	1 I liked undertaking the training programme.					
Implementation	2 The training programme and resources enabled me to administer the TMC confidently.					
Practicality	3 The training programme sufficiently addressed the practicalities of administering the TMC in the childcare setting.					
Implementation	4 I found that the training programme was time efficient to undertake.					
Acceptability	5 The training programme did not require much effort to undertake.					
Implementation	6 The training programme and resources have helped me to detect children with developmental delay.					
Integration	7 The training programme made it clear to me how the TMC can be utilised in the childcare/early learning setting by early childhood educators to facilitate identifying children with mild developmental delays.					
Acceptability	8 It is within scope of practice for an early childhood educator to learn how to use the TMC through the training programme to assess children for developmental delay					
Practicality	9 The training programme and resources have helped me to assess a child in an efficient manner.					
Integration	10 I will be happy to refer to the TMC resources to refresh myself on using the TMC to assess children at my childcare/early learning centre in the future.					
Demand	11 I would recommend others at my childcare/early learning centre to complete the training programme on how to administer the TMC.					
Integration	12 The training programme can be used immediately (it does not need to be modified) to allow early childhood educators to use the TMC to assess children.					
Integration	13 At our childcare/early learning centre, minimal changes would be required from a system level for early childhood educators to use the TMC to assess children on an ongoing basis.					
Acceptability	14 When assessing a child, the TMC is challenging to use even after completing the training programme.					

13 What problems did you encounter when using the TMC to assess children after you had completed the training programme?
14 Do you have any suggestions to improve the training programme?

Thank you for your time in completing this survey.

## Appendix 2 Physiotherapist Reflections

### Physio 1 Reflection

Insights

1 Some transfer of knowledge on how to execute some items of the TMC did not occur from video viewing only for all ECEs.

Suggestions/potential solutions

To add a live TMC demonstration with commentary during training or perhaps although video is being viewed during the training, pauses should be made to direct the viewers to specific techniques and instructions used by the examiner

For example:

All items:

- Verbal cueing (often less or less complex is best, see what child does with minimal instructions first, particularly verbal cues for head righting task would be helpful)
- Observe child from start if they demonstrate the task automatically no need to redo formally.

Eye follow:

- Notice at the speed that the examiner is moving the toy.
- Notice the directions side to side (horizontal), up and down (vertical) and circular both (clockwise then anticlockwise).
- Notice the range/distance/size of toy movement (the size of a steering wheel).
- Notice that the toy is at the child’s eye level not your eye level.
- Notice how you angle the toy’s face or focal point towards the child in an arc.
- Note: Be aware of glare esp. if conducting outside.

Head righting

- Watch for automatic response.

Fine Motor: Light touch localisation

- Notice important fingers for this age: thumb, index and middle fingers.
- Notice how touch is administrated: light and 1 point not a sweep action.

Forward/backward line jump

- Notice there is no pause you demonstrate with no pause also.

Ball catch

- Notice throws underarm.
- Notice distance approx. 2 m away but usually double the height of the child away so smaller shorter you will be closer.

2 ECEs are keen for more knowledge about the interpretation of results and how they can provide changes to how they interact with children and modify teaching style to cater to the specific needs of each child.
3 ECEs worried about head righting—lifting the child and instructions to use.
4 Score sheet—Add scoring options for each item and describe expected response.
5 Manual language is a bit too technical for staff and needs to be simplified.

### Physio 2 Reflections

Training reflections

- Photo of one of the hand positions in TMC manual does not align with the photo in videos.
- Recommended order suggested at start of demo video is different to the chronological order of items on the score sheets. This is probably fine but clarify in the video that the recommended order is for efficiency purposes only.
- Concerns about head righting and lifting heavy children or staff with musculoskeletal issues. Any alternatives? (slight modifications can be made). Also, Educators wanted to know what instructions to use with children when performing this task, e.g., for eyes open, Educator to ask the child to focus on something and to stand tall/hold body straight to avoid tendency to just flop/be silly.
- English as an additional language children may not be able to do items due to language difficulties. Because it is not a norm‐referenced test, more than one demo and practice can be given.
- Recommend pausing in demo video around items to clarify. This would be particularly useful with the latter items because the latter items are more challenging and educators are not as familiar with them. Pausing would help staff to better digest the info too.
- ASD children—take longer, but this is atypical in itself.
- Consider labelling items on the video with their number.
- Consider adding a physical demo of the TMC as part of the training package.
- You cannot hear the common verbal cues we use in the TMC, but then our language is different to educators, and educator language can be just as effective.
- Manual language is still a bit too complex for staff.
- Not clear from demo video or manual if, for tactile, do one hand first or both hands at the same time.

TMC assessment reflections

- Difficult for staff because they need staff relief.
- Would be better if in a format where can easily come back to or can do multiple children on an item at once ie via an app.
- A lot of staff found that when using TMC in their 4× assessments, doing two children at once or having children in the background was very distracting for them and the child (might be different once they’ve used it more).
- Pro of teacher is that if child does not perform at their best (e.g., tired or sick) then if they have seen the child do the item at a higher level, i.e., in the am, then they can score based on that (this would work for fine motor items).
- Staff not familiar with instructions/verbal cues to use but educators' language just as effective.
- Lots of feedback was needed for staff for ocular motor and tactile (e.g., for ocular motor, use an arc approximate size of a steering wheel; for tactile, do not stroke or vibrate).
- Staff needed the physio support for at least the first couple of assessments.
- Score sheets—It would be useful to have on the score sheet for each item: What it is scored out of, i.e., −1,0 or 1; it would also be helpful to have a few more prompts about the age‐appropriate response, e.g., how long need to stand on each leg to get a 1 (4–8 s), how many hops need to complete on each leg to get a 1 (5 or more).

General

- Educators want to understand what the TMC results mean and how they can provide immediate support to an ‘at‐risk’ child within the childcare setting based on the child’s strengths/areas for improvement.

## Appendix 3 Mapping of Bowen et al.'s Feasibility Framework* Used in This Study With Quantitative and Qualitative Questions

**Table:**

Domain	Operationalisation of the domain for the purposes of this study	Mapping of domain to quantitative questions	Mapping of domain to qualitative questions^a^
**Acceptability**	Satisfaction with the TMC training and use of the TMC tool	*I liked undertaking the training programme*. *The training programme did not require much effort to undertake*. *When assessing a child, the TMC is challenging to use even after completing the training programme*.	*What problems did you encounter when using the TMC to assess children after you had completed the training programme?* *Do you have any suggestions to improve the training programme?*
	The intention for ongoing use of the TMC tool	*I will be happy to refer to the TMC resources to refresh myself on using the TMC to assess children at my centre in the future*.	
	The fit of the TMC tool within the organisational culture of the early childhood care centre.	*It is within the scope of practice for an ECE to learn how to use the TMC through the training to identify children at‐risk of DD*.	
	The perceived appropriateness of the TMC tool.	*The training programme made it clear to me how the TMC can be utilised in the childcare/early learning setting by early childhood educators to facilitate identifying children with mild developmental delays*.^*b*^	
**Demand**	Perceived demand for the TMC training and TMC tool for self and others.	*I would recommend others at my childcare/early learning centre to complete the training programme on how to administer the TMC*.	
	The actual use of the TMC training and TMC tool.		
	Perceived demand for further training.		
**Implementation**	Degree of success of the TMC training to enable use of the TMC tool by educators.	*The training programme and resources have helped me to detect children with developmental delay*. *I found that the training programme was time efficient to undertake*.	
	Efficiency, speed or quality of using the TMC tool	*The training programme and resources enabled me to administer the TMC confidently*.	
**Practicality**	Ability of educators to use the TMC tool after undertaking the training with the existing resources, time and/or commitments	*The training programme sufficiently addressed the practicalities of administering the TMC in the childcare setting*. *The training programme and resources have helped me to assess a child in an efficient manner*.	
	The perceived fit of the TMC training and TMC tool within the existing infrastructure of the centre.	*The training programme made it clear to me how the TMC can be utilised in the childcare/early learning setting by early childhood educators to facilitate identifying children with mild developmental delays*.^*b*^ *The training programme can be used immediately (it does not need to be modified) to allow early childhood educators to use the TMC to assess children*. *At our childcare/early learning centre, minimal changes would be required from a system level for early childhood educators to use the TMC to assess children on an ongoing basis*.	
**Integration**	Perceived sustainability of the TMC training and TMC tool.	*I will be happy to refer to the TMC resources to refresh myself on using the TMC to assess children at my childcare/early learning centre in the future*.	

*Note:* *Bowen DJ, Kreuter M, Spring B, Cofta‐Woerpel L, Linnan L, Weiner D, et al. How we design feasibility studies. Am J Prev Med. 2009;36(5):452‐7.

Abbreviation: TMC, targeted motor control.

^a^ Qualitative questions were constructed specifically so that responses could apply to any of the domains.

^b^ This question was relevant to Acceptability and Practicality domains.

## Appendix 5 Improvements Suggested for the Training

- Physiotherapist 2 (Sydney) ‘You can’t hear the common verbal cues we use in the TMC, but then, our language is different to educators and educator language can be just as effective’.
- Educator 11 (> 10 years' experience, Perth) ‘Make it clear that more than 1 demo can be given eg. children where English is an additional language’.
- Physiotherapist 1 (Brisbane) ‘Manual language [is] a bit too technical for staff and needs to be simplified’.
- Physiotherapist 2 (Sydney) ‘… pausing in demo video around items to clarify … [as] latter items are more challenging’.
- Educator 11 (> 10 years' experience, Perth) ‘During the training it would have been beneficial to watch item on scoring video, score on individual sheet and then discuss result and reasoning before watching next item’.
- Educator 1 (> 10 years' experience, Brisbane) ‘It was great to have a go at a scoring video. [Consider] labelling items on the scoring video with their [corresponding] number on the TMC score sheet’.
- Educator 9 (> 10 years' experience, Perth) ‘… other short videos going into some of the details of each task [would be helpful]’.
- Physiotherapist 1 (Brisbane) ‘.…whilst the video is being viewed during the training, pauses should be made to direct viewers to specific techniques used by the examiner’.

Improvement suggested for the TMC tool.

- Educator 10 (3–5 years' experience, Perth) ‘The assessment form would benefit from having a numbered scale to assist in identifying which items are scored 0‐1 and ‐1 to 1’.
- Educator 4 (> 10 years' experience, Brisbane) ‘It would be good to have the required numbers for each activity that the child needs to meet’.
